# Mindfulness and Subjective Well-Being of Indian University Students: Role of Resilience during COVID-19 Pandemic

**DOI:** 10.3390/bs13050353

**Published:** 2023-04-24

**Authors:** Teena Bharti, Nidhi Mishra, Satish Chandra Ojha

**Affiliations:** 1 Indian Institute of Management Bodh Gaya, Bihar 823234, India; 2 School of Entrepreneurship and Management (formerly STEP-HBTI), Harcourt Butler Technical University, Kanpur 208002, India

**Keywords:** COVID-19, pandemic, coronavirus, mindfulness, resilience, subjective well-being

## Abstract

The coronavirus (COVID-19) pandemic is presently a global health issue that negatively affects the mental health and well-being of students globally. The latest investigations have recognized the role of mindfulness in individual subjective well-being. This study explores the mediating role of resilience in the overall relationship between mindfulness and subjective well-being among Indian university students during the COVID-19 pandemic. The data was collected between 10 August 2020 to 24 October 2020 via a self-administered questionnaire from 589 university students in India. Results revealed that resilience has a partial mediating role between mindfulness and subjective well-being. The results substantiate that resilience has an important role in mindfulness, exercising its advantageous effects on mental health of the students in higher education institutions. This research adds to the knowledge base of mindfulness and subjective well-being of university students, especially in contingent times. Lastly, the study contributes to the existing mindfulness theory.

## 1. Introduction

Psychological research has concentrated on how to sustain individuals’ mental and physical health and lessen the adverse consequences of modernization [1]. The term mindfulness is intellectualised as a malleable state of individual awareness involving enhanced attention and non-judgemental understanding of external as well as internal events as they occur [2]. This concept of mindfulness has been drawn from Buddhist literature and is defined as “the ability to pay attention in a particular way–on purpose, to the present moment, nonjudgmentally” [3,4]. Mindfulness is defined as “a state in which one is able to give uninterrupted attention over a period of time in a non-judgmental way to ongoing physical, cognitive and psychological experience, without critically analysing or passing a judgment on that experience” [5]. Mindfulness is a psychological trait that enhances the emotional and cognitive regulation [6]. Furthermore, mindfulness is considered as “paying attention to a mode of consciousness that signifies presence of mind” [7]. Research has suggested that mindfulness is a “resourceful construct” which is described as a “practicing technique and skill” [2]. Alternatively, mindfulness serves as a resource that provides the ability to cope with difficult situations and problems. However, mindfulness is a skill that needs to be developed and practiced to avail the benefits associated with it [4].

Previous research has revealed high correlation between various measures of mindfulness and measures of subjective well-being [8]. Numerous researchers [9,10] have recognised that mindfulness-based techniques, such as yoga and meditation, lead to an increase in individuals’ well-being. A few studies [11], conducted on a sample comprising college students, have reflected that mindfulness may influence subjective well-being via different mediating variables, namely self-esteem, core self-evaluation, optimism, and emotional intelligence. After traversing the literature on mindfulness and subjective well-being, this research surmised that resilience may affect the liaison between mindfulness and subjective well-being in students in the context of the COVID-19 pandemic. Higher educational institutions (HEIs) need to exhibit resilience to withstand the shock of the pandemic and maintain continuity in their core activities by addressing issues related to potential socio-psychological damages to various stakeholders, while adapting to the new normal [12]. COVID-19 has affected everyone, being a significant source of stress, anxiety, and other related psychological disorders [13], and still served as a key source of opportunity and innovation [14]. A longitudinal study conducted on a sample of university students in China showed that growth in novelty-seeking behaviour of students was associated with observed decrease in stress, anxiety, and depression during the lockdown period of the COVID-19 pandemic [15]. This finding supports the rationale of the current study, since novelty seeking is an important dimension of mindfulness, whereby an individual actively notices new things and flexibly responds to the context [16]. Exploring new and varied activities and thinking in a non-linear and creative manner (cognitive flexibility) are a few attributes of a mindful person which can contribute significantly towards subjective well-being of individuals in times of crisis, such as the COVID-19 pandemic [17].

Several studies have reported on the impact of COVID-19 in HEIs in India [18]. These studies show that Indian HEIs have been quick to react to the crisis created by the pandemic situation by adopting remote working and online teaching-learning activities [19]. The pandemic provided an opportunity for HEIs to develop new pedagogical approaches and tools to continue providing teaching, research, and service to students [20]. In the Indian context, the University Grants Commission (UGC) and Ministry of Human resource Development (MHRD) launched virtual platforms for online depositories, e-books, and other online teaching material [21]. While this agility exhibited by HEIs has ensured continuity of teaching-learning processes, it has also significantly impacted the well-being of the students and employees associated with these institutions [22]. However, not all individuals are similarly impacted by such adverse situations, and the magnitude and valence of impact are likely to depend on the individual traits of the students and employees. In the current study we consider two such individual traits—mindfulness and resilience—and study their impact on the subjective well-being of students studying in Indian HEIs. In the present research, we try to explore the role of resilience as the mediating variable in the association between mindfulness and subjective well-being.

The theoretical background of the study lies in the mindfulness theory, which focuses on the context awareness in the present moment. It is based on the principle of keeping a receptive mind to alternative categories and perspectives [23]. It suggests that automatic behaviour, having a singular perspective, could make it hazy difficult to see the entire picture, and hence, influence the relationships and overall performance [24]. It suggests that having more self-acceptance would help in creating a clear vision [21] and an ability to bounce back, thus expanding the narrow categorization [19] that affects well-being and stress [25,26]. Henceforth, by examining the role of resilience, this study will add to the active learning base in mindfulness and subjective well-being, especially for university students. Keeping the above in mind, this research has the following objectives:

To test the proposed research model for a first order model or a second order model.

To explore the mindfulness and subjective well-being relationship in Indian university students, especially during the COVID-19 pandemic.

To explore the mindfulness and resilience relationship in Indian university students, especially during the COVID-19 pandemic.

To explore the mediating role of resilience in mindfulness and subjective well-being relationship in Indian university students, especially during the COVID-19 pandemic.

## 2. Literature Review

### Resilience, Mindfulness and Well-Being

Resilience is a personality trait that helps an individual to deal with adverse situations and achieve decent adjustment and development during the entire process [27]. Resilience is portrayed as an individual’s capacities, assets, and aptitudes needed to adjust to the distressing and unfavourable circumstances, and to conquer requesting circumstances [28]. Resilience is also defined as a trait that immunizes individuals to adversity and traumatic events [29]. Furthermore, resilience can be understood according to two different schools of thought [30]. According to one, resilience is considered a personality trait that is quite stable, while the other considers it a capacity which can be changed after some time, contingent upon the circumstance [31]. Further, resilience has also been conceptualized as a dynamic process by some researchers, thus highlighting the importance of cultivating resilience in the face of anxiety and mental health problems [32]. Resilience is also defined as a skill to maintain high well-being when an individual encounters life hardships [33]. It is resilience that helps us understand how some individuals can withstand and thrive under the pressure they experience in their lives while others cannot. Resilient individuals can endure their psychological as well as physical health both by cushioning the negative consequences [34] and by cultivating mental well-being [33]. Therefore, resilience is imperative to enhancing the subjective well-being.

According to a study, mindfulness and an accepting orientation towards experience facilitates the prevention of depressed thinking, thus fostering psychological resilience [35]. furthermore, mindful individuals are resilient in nature, as they tend to engage comparatively less in contemplation and chronic worrying, but rather sustain a problem-solving attitude [36]. As per another researcher, mindfulness determines the possibility of nurturing resilience, as mindful individuals are better able to comprehend challenging situations and respond accordingly, instead of reacting without due deliberation [37]. These individuals are receptive to new perceptual classifications, have a propensity to be more innovative, and may deal with complex situations in more resilient manner without becoming overwhelmed or shutting down to the exterior world [38]. The idea is that, when stimulus is strong enough, attention is engaged, which is exhibited as turning towards the object. This is well explained by the science of neuroscience offering understanding into the mechanisms of mindfulness through resilience. The art of being present in the moment through mindfulness can break the pattern of negative thought processes that keep people obsessing about past failures and challenges. It helps in strengthening the association between the amygdala and the prefrontal cortex, promoting a self-control that will prevent people sliding into negative thoughts and setbacks [39]. Thus, mindful individuals, even when they are struck with setbacks and failures, are able to exhibit resilience such that they bounce back easily and are able to maintain well-being during challenging circumstances.

**Figure 1:**
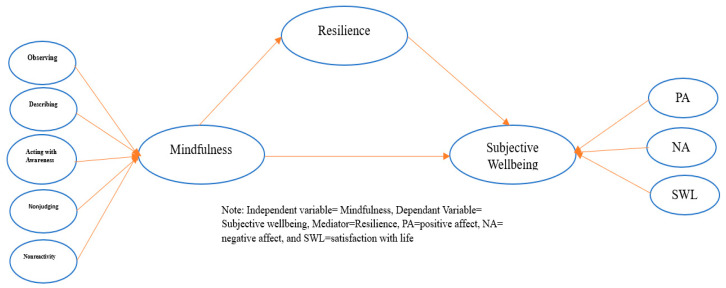
Proposed Model for research.

Referring to the extensive literature base on mindfulness, it has been reflected that resilience is an outcome of mindfulness [39,40] and in turn affects the life satisfaction and affect in individuals [13,41,42,43]. The mediating role of resilience in the association between mindfulness and variables of well-being have been evidenced by a number of studies in the literature. An undergraduate study found that resilience partially mediates the mindfulness and subjective well-being relationship [44]. Another study on a sample of firefighters and law enforcement individuals suggested the partial mediation of resilience between mindfulness and burnout, such that improved mindfulness was associated with improved resilience, and consequently decreased burnout [8]. Additionally, substantial evidence on a sample of college students highlighting the mediating role of emotional resilience in the association between mindfulness and emotional regulation has been found [45]. The current study seeks to broaden the above literature, reflecting the role of resilience as a mediator in the mindfulness and subjective well-being association in the context of the COVID-19 pandemic. Explicitly, an individual is likely to reflect higher levels of mindfulness resulting in higher level of resilience, and thereby increased levels of subjective well-being. The proposed model of the current research is presented in Figure 1.

HEIs need to exhibit resilience to withstand the shock of the pandemic to maintain continuity in their core activities by addressing issues related to potential socio-psychological damages to various stakeholders, while adapting to the new normal [12].

Studies suggest that the vital indicators of an individual’s mental health are mental health indicators such as fear, confused thinking, extreme mood swings, etc., but these are often side-lined by people [39]. In the times of COVID-19 pandemic, the world is witnessing a rapid shift and constant change, subject to the environment. Owing to the fear of contagious and pathogenic nature of the virus, it has led to increased levels of anxiety, stress, nervousness, and depression among individuals. Furthermore, many university students suffer from mild depression and mild anxiety. Additionally, the study established that both depression and anxiety are found more in females [46]. In the pandemic times, resilience owing to the environment provided by universities/institutions has been considered as an asset that provides constant support to students’ mental as well as emotional health requirements [47]. During the degree courses, university students often experience many issues regarding mental health and well-being in comparison to their batchmates from a non-university background [48]. Considering this, resilience is predominantly significant, as student life can be rather challenging and intricate, demanding the competence to survive challenging situations such as a highly competitive curriculum, financial difficulties, study-life balance, and interpersonal relationship glitches. Consequently, the present research might give some insights into the probable psychological machinery for improving students’ subjective well-being at university. Based on the above literature the following hypotheses are drawn:

**H1.**  *Mindfulness is positively related with subjective well-being in Indian university students, during the COVID-19 pandemic.*

**H2.**  *Mindfulness is positively related with resilience in Indian university students, during the COVID-19 pandemic.*

**H3.**  *Resilience mediates the relationship between mindfulness and subjective well-being in Indian university students, during the COVID-19 pandemic.*

## 3. Psychological Well-Being of University Students in COVID-19 Times: Rationale behind the Study

This study is even more pertinent in the context of the COVID-19 pandemic, which is marked by volatility, uncertainty, complexity, and ambiguity (VUCA) [49]. These characteristics of the current pandemic have resulted in unprecedented demands and challenges for both universities and students alike. This has raised concerns among the universities as to how they could restructure their systems and process so that they are agile and sustainable in a VUCA environment [50]. The scope of the current study, however, is focused on how student’s responses to uncertain and dynamic environments could be enhanced so that their subjective well-being is not compromised. Studies have reported the evidence of stress among students in higher education due to negative effects of the pandemic on outcomes, such as delayed graduation, loss of job, internship or a job offer, and lower expected salaries [51]. The effect of the COVID-19 pandemic on students’ outcomes and expectations is, however, heterogeneous, and varies systematically with existing socioeconomic factors such as income levels, and the mediating effect of the magnitude of financial and health shocks experienced [48,51]. While financial shock was operationalized as loss of job for the student, loss of income for student’s family members, or whether the student will fail to repay debt, health shock was operationalized as the student’s subjective health assessment or chances of contracting COVID-19 and being hospitalized [52]. The current study argues that the effect of the COVID-19 pandemic on students could further vary depending upon the psychological and cognitive resources they possess such as mindfulness and resilience. These resources could buffer the impact of challenges created by the external environment by impacting how the individual responds to the external demands; this can in turn help them to either enhance or at least maintain their subjective well-being. Moreover, the results of the current study can be applicable to any other crisis marked by VUCA, such as economic recession, mergers, and acquisition, disruptive or dynamic environment, etc.

## 4. Materials and Methods

### 4.1. Participants and Procedure

The study utilized a quantitative online survey research method for collecting the online responses from undergraduate university students in India. The duration of data collection was over an 8-week period (10 August 2020–24 October 2020). Owing to the ongoing first wave of the COVID-19 pandemic in India at the time of data collection, the response rate was approximately 30%, with 589 complete responses. After collecting the responses, we conducted a thorough screening to eliminate the incomplete responses and outliers. We eliminated 44 responses because of missing values that lead to incompleteness and non-usability. This study collected a usable sample (total responses received from voluntary participation of 589 undergraduate university students (436 male, 153 female)) with age ranging between 18–23 years with an average age of 20 years (SD = 1.23). Thus, this process was in line with the guidelines of structural equation modelling (SEM) [53]. The questionnaire also included a demographic details section. The participants willingly provided consent to participate in the online survey and were assured of the complete confidentiality of their data.

### 4.2. Measures

#### 4.2.1. Mindfulness

The study used a “five-facet mindfulness questionnaire (FFMQ) [54] to measure the trait mindfulness. This scale consists of 39 items assessing the five subscales: observing, describing, acting with awareness, nonjudging of inner experience, and nonreactivity to inner experience. The items were measured on a five-point Likert scale (5 = always to 1  =  never). The sample items of this scale are “I’m good at finding words to describe my feelings.” and “I tell myself I shouldn’t be feeling the way I’m feeling”. The Cronbach’s α coefficient for all five subscales in this study were 0.82, 0.83, 0.91, 0.79, and 0.77, respectively. The overall reliability coefficient for mindfulness scale was 0.87 [40].

#### 4.2.2. Subjective Well-Being

The subjective well-being of the university students was measured by using Life satisfaction [55,56] and Positive Affect and Negative Affect scale (PANAS) [57].

Life satisfaction.

The SWLS comprises of five brief items [55,56]. A 7-point Likert scale was used, where 1 signalled ‘strongly disagree’ and 7 signalled ‘strongly agree’, to collect responses from the undergraduate university students. The scale included items such as “In most ways my life is close to my ideal” and “I am satisfied with my life”. Researchers suggested that the scale has good psychometric properties with an internal consistency of 0.93 [58,59].

#### 4.2.3. Positive Affect and Negative Affect

The scale to measure positive affect and negative affect (PANAS) consists of 20 adjective words, i.e., 10 words each for negative and positive affect [57], with a Cronbach’s alpha of 0.92 and 0.91. The participants were requested to specify how frequently they usually experienced the negative and the positive emotions on a 7-point Likert scale where the response of 7 indicated “strongly agree” and 1 indicated “strongly disagree”.

#### 4.2.4. Resilience

A short-abridged version of the Connor–Davidson resilience scale (CD-RISC) developed by Connor and Davidson, having 10 items such as “able to adapt to change”, “can stay focused under pressure”, and “not easily discouraged by failure” [60], was used. Items were rated on a 7-point Likert scale that ranges from 1 (not at all) to 7 (very true nearly all the time). The scoring of the scale indicates that higher scores reflect higher resilience. The scale reflects good internal consistency 0.92 [61].

All the instruments used for collecting responses exhibited exceptional internal consistency, test–retest reliability, and convergent as well as discriminant validity.

## 5. Data Analysis

Anderson and Gerbing suggested a two-step mediation process [62]. This study analysed the measurement model to assess the various latent variables as characterized by its indicators using SPSS AMOS 25. The study calculated the model fit criteria in line with the recommendations, i.e., chi-square statistics of less than 3.0; RMSEA (root-mean-square error of approximation) of 0.06 or less; goodness of fit index of 0.08 or more; non-normed fit index (NNFI) of 0.90 or more; comparative fit index (CFI), best if above 0.90; and normed-fit index of 0.09 or more [63].

This research utilised 5000 bias-corrected bootstraps under the bootstrapping procedure with a confidence interval (CIs) of 95%. This process involves continuous resampling from the empirical sample to resemble the original sampling process. The researchers recommended that an indirect role was significant if zero was not included in the computed confidence intervals [64].

## 6. Results

### 6.1. Preliminary Analyses

This section discusses the descriptive statistics, reliability estimates, and correlations between all the variables considered in this study (refer Table 1). Since the data was collected by the self-reported questionnaire method, we also tested for the presence of CMV (common method variance). A Harman single factor test was used, and it found that the variance explained by common factor was not more than 50 percent [65].

### 6.2. Pooled Confirmatory Factor Analysis

This section deals with the measurement model as stated in objective 1 of the study, i.e., pooled/combined confirmatory factor analysis of the first as well as second order mod-el of study variables (mindfulness, resilience, and subjective well-being). The results con-sisted of M1 = one factor model with 74 items; M2 = two factor model with mindfulness and SWB; M3b = first order model with five reflective dimensions (mindfulness, resilience, positive affect, negative affect, and satisfaction with life); and M3a = second order model of mindfulness, resilience, and subjective well-being, i.e., first order model with five main factors (mindfulness, resilience, life satisfaction, positive affect, and negative affect) and second order model with three main constructs (mindfulness, resilience, and subjective well-being). The results of both M3b and M3a met the recommended criteria suggesting a very satisfactory fit. However, the second order model (M3a) reported a slightly better fit index: χ^2^/df = 2.315, GFI = 0.895, CFI = 0.957, NFI = 0.917, NNFI = 0.939, SRMR = 0.050 and RMSEA = 0.045, and reflected an overall improved fit as depicted in Table 2. The factor loading for all the latent variables was reliable (*p* < 0.001), indicating that all the latent factors (factors/variables that can only be inferred indirectly from other observable variable) were well implied by the indicators.

The results depicted in Table 3 displayed that the values of indicators of convergent validity, namely, composite reliability (mindfulness = 0.865, resilience = 0.853, SWB = 0.837) and average variance extracted, i.e., AVE (mindfulness = 0.679, resilience = 0.661, SWB = 0.633) were greater than the threshold values (CR > AVE and CR > 0.70) [66]. Additionally, the discriminant validity of factors (mindfulness = 0.875, resilience = 0.825, SWB = 0.813) were confirmed, with the values of MSV (mindfulness = 0.233, resilience = 0.261, SWB = 0.261), ASV (mindfulness = 0.207, resilience = 0.245, SWB = 0.221) and AVE (mindfulness = 0.679, resilience = 0.661, SWB = 0.633) satisfying the suggested criteria, i.e., MSV < AVE and ASV < AVE [66].

### 6.3. Testing the Hypotheses

Hypotheses (1–2) of theoretical model.

The results shown in Table 4 indicate that: there was a positive and significant rela-tionship between (a) mindfulness and resilience, b = 0.521, *p* < 0.01, (CI; 0.113, 0.251); (b) mindfulness and well-being, b = 0.121, *p* < 0.01, (CI; 0.122, 0.273); and (c) there was a posi-tive and significant relationship between resilience and well-being, b = 0.495, *p* < 0.01, (CI; 0.129, 0.283). Therefore, Hypotheses 1 and 2 were supported.

Mediation effect.

This research tested the mediation link (H3) between the independent and the out-come variables via the mediating variable [67]. The alternative models for partial media-tion were compared with the full mediation model in this study. The partial mediation fit indices (χ^2^/df = 2.11, RMSEA = 0.032, CFI = 0.96; NFI = 0.94, GFI = 0.95, NNFI = 0.96, AIC = 2498.53) were better as compared to the full mediation model, though the values for the full mediation model were also within the threshold limit (χ^2^/df = 2.75, RMSEA = 0.06, CFI = 0.98; NFI = 0.93, GFI = 0.94, NNFI = 0.94, AIC = 2819.13). Additionally, the χ^2^ test of differ-ential reflected significant outcomes (Δχ^2^ (2) = 5.25, *p* < 0.01) and depicted that the partial mediation model was most suitable for hypothesis testing. Additionally, the partial medi-ating model with control variables was also tested to compare the results, which suggested acceptable fit indices (χ^2^/df = 2.14, RMSEA = 0.046, CFI = 0.94; GFI = 0.92, NFI = 0.93, NNFI = 0.92, AIC = 3869.12). Further the standardized effects of control variables on the variables currently studied were not significant and acceptable. Hence, the analysis reflects that the partial mediation model, excluding the control variables, was deemed fit for hypothesis testing. In addition, Table 4 reveals the mediation findings, indicating direct, indirect, and total effects for the entire sample. The positive indirect path was significant (MF Res SWB) was significant (i.e., b = 0.257, 95% CI [0.221, 0.391]).

**Figure 2:**
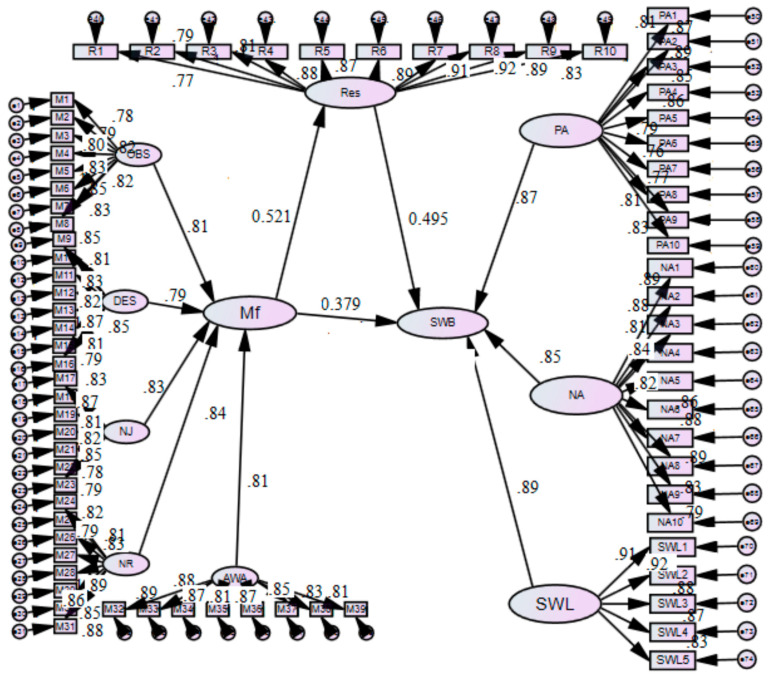
Structural Path model of the research. Note: MF = Mindfulness, Res = Resilience, SWB = subjective wellbeing, PA = positive affect, NA = negative affect, SWL = satisfaction with life, OBS = Observing, DES = Describing, AWA = Acting with Awareness, NJ = Nonjudging, NR = Nonreactivity.

Table 4 represents that the overall indirect effect of mindfulness on well-being via resilience was significant, with b = 0.257, accelerated CI [0.221, 0.391] and 95% bias correction. Additionally, the total effect of mindfulness on wellbeing is significant, with b = 0.379 CI [0.317, 0.475] and 95% bias correction. Therefore, based on the results, resilience can be said to act as the mediating variable between mindfulness and well-being. The depiction of the structural path model is provided in Figure 2.

Hence, we can say that all the objectives of the study have been achieved and the hypotheses have been verified.

## 7. Discussion and Implications

This research aimed to analyse the contribution of mindfulness towards subjective well-being and further develop the existing knowledge base by examining the mediating effect of resilience between mindfulness and subjective well-being in the context of the COVID-19 pandemic. The results of the statistical analysis revealed that all the hypotheses were supported. This is in line with past studies [68,69,70,71,72], reflecting that mindfulness was positively related with individual well-being. In 2014, Sharna et al. [73] reported that effective resilience and stress management reduces depression and anxiety and leads to improved wellbeing of the individual. This comes in the presence of mindfulness which has beneficial effects on various indicators of mental health such as reduced anxiety and depression, and increased compassion, wellbeing, and resilience [39,74]. Even though some preceding studies have analysed the mediating role of resilience in the relationship between mindfulness and well-being [74,75], there is not enough exploration that has probed this mediating role in times of crisis or uncertain times, such as the COVID-19 pandemic situation. The results of the current study have strengthened the study predictions.

The theoretical foundation for the present research emphasises that the “acknowledgment” and “awareness” attributes of mindfulness facilitate the advancement of greater resilience [76,77]. This further aids optimism, enthusiasm, and the perseverance characteristics of resilient individuals, and may lead to greater well-being [78,79]. Observing and pausing the mind may help endure being dragged into a setback. Additionally, mindfulness is a phenomenon processed by the prefrontal cortex, which is responsible for more connectivity in the brain and is held responsible for stunting the negative affect and consequently increasing the positive emotions in individuals by reducing the overactivation of amygdala involved in experiencing significant emotions [80,81]. An increased level of mindfulness aids emotional balance and benefits mental health [82,83]. Thus, mindful people have better psychological adjustment abilities and decision-making skills as they are consciously cognizant of their vicinity [84]. They are open to the changes, reflect creativity, and can better cope with stress, anxiety, or negative emotions [85]. These results emphasize the importance of resilience in the relationship between mindfulness and subjective well-being, suggesting that resilient individuals often take charge of the situation by changing the overall narrative [86]. The study thus establishes a relationship between mindfulness, resilience, and subjective well-being. Resilience serves as one of the potential mechanisms by which mindfulness impacts well-being. Thus, mindfulness-based interventions and training could enhance personal resources such as optimism, flexibility, patience, and self-acceptance, resulting in overall health and well-being.

The study signifies the relevance of mindfulness and resilience in enhancing the well-being of students. Traditionally, students have always thought of social media as a portal to put forward their ideas on issues which make them jovial and happy [87,88]. It was never a platform for learning, but when COVID-19 suddenly hit the world it created a catastrophe for society at large. The student fraternity, overnight, found themselves in a dilemmic position. The platform which was more for their leisure and pleasure had suddenly become a platform for learning [89]. As a result of this sudden shift, students suffered at large, as their body was not accustomed to this change and it resulted in increased screen time, which ultimately led to several physical and mental health disorders [90]. The findings of the current study suggest that universities should invest resources in promoting mindfulness and resilience among its students, through interventions such as life-skill courses and co-curricular activities that will help them cope with academic pressures as well as ensuing environmental change, thus fostering well-being [91]. This study recommends that universities should use mindfulness-based interventions to promote resilience, keeping in mind that university students face significant stress which may be due to the curriculum or changing patterns of education, along with anticipation regarding the future. Mindfulness acts as a buffer in coping with the increased stress levels. Furthermore, the results support earlier studies suggesting that mindfulness and resilience can be useful for understanding the mind and the brain, and, hence, alleviating issues related to stress, anxiety, depression, etc.

## 8. Limitations and Future Scope

This research has some limitations. Firstly, the data is based on a self-reported questionnaire, which are often subject to biases, owing to social desirability. Secondly, this study had a cross-sectional design, and future research can consider longitudinal/experimental design to establish the causal relationship between the variables. Thirdly, the study can consider emotional intelligence and cognitive flexibility as means to mindfulness. Future research might also explore the possible mediating and moderating role of other variables, such asCOVID-19, trust, interpersonal relationships, demographic variables (income, age, gender, and others), social interventions, coping mechanisms, etc., between mindfulness and well-being. Additionally, the multifaceted nature of mindfulness can be explored in future research along with other outcome variables such as PsyCap, happiness (life satisfaction, positive affect, etc.), and their sub-dimensions. Furthermore, future research directions should include the comparative analysis between different instruments of mindfulness and subjective well-being to establish the concrete relationship. In addition, a comparative analysis can also be conducted to explore whether there are different approaches to implement mindfulness interventions during or after the pandemic, or whether mindfulness can be implemented in the future through online or offline methods.

## 9. Contributions

This research has several contributions. This study provides a vivid account of literature that would be helpful/beneficial for the higher educational institutions. The study adds to the existing literature on mindfulness, resilience, and subjective well-being, especially in times of crisis. The current study’s results can be compared with the earlier existing research because of the similarity in the sample (university students in India) and similar independent, dependent, and mediating variables [44]. However, while the earlier study was conducted in a pre-COVID scenario and in a classroom setting, the current study was conducted during the COVID era and via an online medium. The findings of the current study show that the strength of the relationship among the variables under study have changed significantly in comparison to the earlier study [44]. The difference can be partially attributed to the changed environmental context due to COVID-19, apart from other factors such as online medium, different university set up, etc. It significantly supports the arguments provided by the different disciplines, namely neuroscience [92], consumer behaviour [93], medicine [5], and psychology [33], that suggest the contribution of mindfulness in developing resilience in stressful and traumatic situations, leading to overall subjective well-being. In another experimental study conducted on adolescent students in China, it was found that mindfulness training intervention increased students’ resilience and that the rate of increase grew gradually [94]. Moreover, individuals having higher baseline levels of positive personality traits, such as mindfulness, resilience and optimism, were less likely to experience negative mental health consequences of the COVID-19 pandemic in Dutch and Belgian samples [95]. The current study supports these findings by providing a cross-sectional account of the relationship between mindfulness, resilience, and well-being in an emerging economy such as India. Keeping the pandemic in mind, improving the realm of developmental programs such as resilience and mindfulness training will help us to gain insights into understanding how university students can withstand the ongoing change, and yet adapt to make the most of it reasonably.

Further, this empirical research extensively adds to the mindfulness theory supporting its relevance in the changing social concerns, per se changing workplace dynamics and changing education system, and in turn preparing the university students for present, tomorrow and the future [96,97,98]. It suggests that in due course of time, universities and parents need to focus on life skill-enabling courses, such as mindfulness and resilience, which would help students become more resilient and less mindless.

## Figures and Tables

**Table 1:** Descriptive statistics and inter-correlations.

Variables	Mean	SD	Mindfulness	Resilience	SWL	PA	NA
Mindfulness	5.02	1.40	(0.875)				
Resilience	4.49	1.47	0.568 *	(0.923)			
SWL	5.05	1.56	0.439 *	0.431 **	(0.929)		
PA	4.67	1.24	0.354 **	0.431 **	0.338 **	(0.924)	
NA	3.49	1.33	−0.237 **	−0.311 *	−0.398 **	−0.430 **	(0.931)

Note: SD = Standard deviation, PA = Positive Affect, SWL = Satisfaction with Life, NA = Negative Affect, Cronbach’s alpha shown in bracket. * *p* < 0.05, ** *p* < 0.01

**Table 2:** Fit indices results (pooled CFA).

Details	χ^2^/df	GFI	CFI	NFI	NNFI	RMSEA	SRMR
One factor model (M1)	4.560	0.725	0.721	0.717	0.713	0.711	0.191
Two-factor model (M2)	3.209	0.785	0.789	0.792	0.793	0.799	0.101
1st-order model (M3b)	2.537	0.857	0.939	0.901	0.923	0.059	0.062
2nd-order model (M3a)	2.315	0.895	0.957	0.917	0.939	0.045	0.050
Recommended criteria	<3.00	≥0.80	≥0.90	≥0.90	≥0.90	≤0.08	≤0.08

Note: χ^2^ = chi-square; df = degree of freedom; NFI = normed fit index; GFI = goodness of fit index; CFI = comparative fit index; RMSEA = root mean square error of approximation; NNFI = non-normed fit index, SRMR = standardized root mean square residual M1 = one factor model with 74 items, M2 = two factor model with mindfulness and SWB, M3b = first order model with reflective five dimensions (mindfulness, resilience and positive affect, negative affect, and satisfaction with life); M3a = second order model of mindfulness, resilience, and subjective well-being.

**Table 3:** Validity and reliability statistics of the measurement model.

	CR	AVE	MSV	ASV	DV	Mindfulness	Resilience	SWB
Mindfulness	0.865	0.679	0.233	0.207	0.791	-		
Resilience	0.853	0.661	0.261	0.245	0.789	0.479 **	-	
SWB	0.837	0.633	0.261	0.221	0.797	0.433 **	0.509 **	-

Note: ** correlation are significant at the 0.01 level, SWB = Subjective Well-being, AVE = Average Variance Extracted, MSV = Maximum Shared Variance, CR = Composite Reliability, ASV = Average Shared Variance, DV = Discriminant validity.

**Table 4:** Coefficients for direct, indirect, and total effects.

Effect of A on B	Effect Estimates	SE	*p* Value	95% CI	
				Lower	Upper
Direct effect					
Mindfulness → Resilience (a)	0.521	0.03	0.00	0.113	0.251
Mindfulness → SWB (c′)	0.121	0.04	0.00	0.122	0.273
Resilience → SWB (b)	0.495	0.03	0.00	0.129	0.283
Indirect effect					
MF → Res → SWB (ab)	0.257	0.04	0.00	0.221	0.391
Total effect					
Mindfulness → SWB (c)	0.379	0.05	0.00	0.317	0.475

Note: CI = class interval, *p* = probability, MF = mindfulness, Res = resilience and SWB = subjective wellbeing, c = c′ + ab wherein c signifies the total effect c′: A direct effect is simply a direct relationship between an independent variable and a dependent variable in presence of the Mediator; a*b: An indirect effect is the relationship that flows from an independent variable to a mediator and then to a dependent variable; the term total effect is the combined influence of the direct effect between two constructs and the indirect effect flowing through the mediator (c = c′ + a*b). Wherein, c in the relationship between independent variable and dependent variable is the total effect.

## Data Availability

Not applicable.
